# Genetic and dietary salt contributors to insulin resistance in Dahl salt-sensitive (S) rats

**DOI:** 10.1186/1475-2840-7-7

**Published:** 2008-04-08

**Authors:** Marlene Shehata

**Affiliations:** 1 Department of Cellular and Molecular Medicine, University of Ottawa Heart Institute, Ottawa, Ontario, Canada

## Abstract

Insulin resistance has been extensively investigated during the past decade because of its proposed role in initiating a cluster of cardiovascular risk factors including hypertension. Insulin resistance is an inherited genetic trait that precedes hypertension in Dahl salt-sensitive (S) rats, and is not present in Dahl salt-resistant (R) rats. Owing to the co-existence of insulin resistance and salt sensitivity of blood pressure in Dahl S, but not R rats, Dahl S rats are used to elucidate the role of dietary salt as a potential link in exacerbating both phenotypes (insulin resistance and salt sensitivity). In light of available data, examining the impact of dietary salt on insulin resistance in Dahl S rats in terms of salt concentration and duration of exposure helps answer the following question: What percentage of dietary salt and for what duration of exposure would we expect an enhanced insulin resistance in Dahl S rats? This commentary gathers all available research done on insulin resistance in Dahl S rats in an attempt to unravel dietary salt contribution to insulin resistance in Dahl S rats.

## Commentary

Over the past decades, insulin resistance and compensatory hyperinsulinemia have been proposed as the common disorder underlying a constellation of risk factors (hypertension, diabetes, and hypertriglyceridemia) referred to as the metabolic syndrome or syndrome X [1]. Insulin resistance and salt-sensitivity of blood pressure co-exist in Dahl salt-sensitive (S), but not salt-resistant (R) rats which in turn raises the following questions: a) Does insulin resistance in Dahl S rats predict their salt-sensitivity? b) Is insulin resistance in Dahl S rats a genetic abnormality or is it a metabolic consequence of salt-induced hypertension in this model? c) Does salt play a role in enhancing insulin resistance in Dahl S rats as it does with hypertension in this model? d) Does salt exert its action at a particular concentration and duration of exposure?

To be able to address the above questions, Dahl S rats have been examined at various stages (weanlings and fully developed hypertensive rats) and with various dietary salt regimens (low, medium and high salt diet for durations of 1 to 4 weeks and up to 3 months of sodium loading). Weanling Dahl S rats when examined before developing hypertension and prior to sodium loading were found to be significantly insulin resistant as evidenced by their higher plasma insulin levels compared to weanling Dahl R rats [2]. This implies that insulin resistance in Dahl S rats is a genetic trait that is inherited and manifested prior to salt-sensitive hypertension in this model. When fully hypertensive Dahl S rats were examined (Dahl S rats become spontaneously hypertensive after 2–3 months [2], but can develop stable hypertension after 4 weeks of 8% NaCl [3], they remained insulin resistant compared to Dahl R rats [2]. Insulin resistance in hypertensive Dahl S rats is manifested by significant changes in several parameters compared to Dahl R rats. These parameters include: i) higher plasma insulin levels [2,4,5], ii) defective insulin-stimulated glucose uptake by adipocytes [4], iii) higher plasma triglyceride concentrations [4], iv) decreased glucose infusion rate, clearance, and utilization rate, and v) higher hepatic glucose production as assessed by the hyperinsulinemic euglycemic clamp procedure [2,5].

Knowing that Dahl S rats are genetically insulin resistant as evidenced by the presence of insulin resistance in weanling Dahl S rats as well as in fully hypertensive Dahl S rats, it's time to address our third and fourth questions which inquire about the role of salt in insulin resistance and whether a specific salt concentration and/or duration of exposure is required for salt to play a role in insulin resistance in Dahl S rats. Controversial reports in the literature have been documented with some studies proposing no effect of dietary salt in advancing insulin resistance and others proposing a significant role of salt in suppressing insulin signaling and precipitating insulin resistance in Dahl S rats. That's why, it is important to analyze all available data in terms of salt concentration and duration of exposure. Salt loading in Dahl S rats did not further increase their insulin resistance when fed 3% NaCl for 1 week [4], or 8% NaCl for 2 weeks [4], or 2% NaCl for 2 months [2]. Only, when Dahl S rats were fed 8% NaCl for 4 weeks did salt significantly enhanced their insulin resistance by markedly suppressing the glucose infusion rate, utilization rate and glucose uptake by the muscles compared to Dahl S rats fed 0.3% NaCl [5]. This implies that 8% NaCl for 4 weeks is the threshold point for salt to exert a recognizable effect on insulin signaling. Going below 8% NaCl and/or a period of less than 4 weeks would not further enhance insulin resistance in Dahl S rats.

Not only did 4 weeks of high salt diet of 8% NaCl exaggerated insulin resistance [5,6]and contributed to a stable hypertension in Dahl S rats[3], but also, it enhanced oxidative stress [7]and inflammation that was strictly dependant on salt [8]. On the other hand, long term exposure (such as for a period of 10 weeks) to a high dietary salt intake of 8% NaCl triggered an inflammatory response and precipitated renal injury in both Dahl S and R rats [8]. Inflammation was assessed by the overly expressed inflammatory mediators such as the c-jun terminal kinase (JNK). This implies that chronic salt loading by itself triggers inflammation and renal injury even in the salt-resistant Dahl R rat. It also suggests that genetic defects in Dahl S rats, possibly along the inflammatory pathway, might predict their enhanced salt-induced inflammatory reaction after a shorter period of 4–5 weeks of exposure to 8% NaCl compared to 10 weeks of exposure in Dahl R rats.

Salt-induced oxidative stress and inflammation might be the mechanism by which high dietary salt concentration of 8% suppresses insulin signaling and precipitates insulin resistance in Dahl S rats. Inflammatory mediators enhanced by high dietary salt include the tumor necrosis factor-alpha (TNF-alpha) or JNK might possibly interfere with normal insulin signaling via a number of mechanisms including: i) disruption of insulin-induced insulin receptor substrate-1 (IRS-1) and phosphoinositide-3 kinase (PI3K) phosphorylation and cellular distribution, ii) reduction in glucose transporter (GLUT4) expression levels, or iii) reduction in GLUT4 translocation to the plasma membrane [9-11]. Suppressed insulin signaling in Dahl S rats via defects in insulin receptor substrates, or defects in their association to the PI3K, or defects in GLUT4 translocation might explain insulin resistance in Dahl S rats. All of the above possibilities might be mediated via the salt-induced inflammation in Dahl S rats.

While one would expect suppressed insulin signaling in Dahl S rats, Ogihara et al., reported that Dahl S rats had an enhanced insulin signaling manifested by an enhanced tyrosine phosphorylation of the insulin receptor, insulin receptor substrates, activation of PI3K and phosphorylation of Akt compared to Dahl R rats. These effects could be simply attributed to the fact that the authors starved the rats for 12 hours prior to the experiment. It is well documented that 12-hour of fasting upregulated hepatic insulin receptors in Sprague Dawley rats (from which Dahl rats were derived) ([12]. Moreover, fasting enhanced tyrosine phosphorylation of the insulin receptor, insulin receptor substrates, activated PI3K and phosphorylated of Akt in insulin resistant rat models compared to their fed controls [13]. These effects of fasting in insulin resistant models are similar to those observed by Ogihara et al. in 2002 in starved Dahl S rats. It is also worth mentioning that fasting significantly enhanced the insulin signaling pathway in lean versus obese subjects [14]. In agreement with the role of fasting in enhancing the insulin signaling pathway in Dahl S rats is the observation that Dahl S rats had a significant weight loss after 4 weeks of high salt diet compared to normal salt diet or their Dahl R controls [5,6,8]. These observations suggest that defects along the insulin signaling pathway might have been hidden by the effect of fasting in Dahl S rats, which significantly improves insulin sensitivity [15]. It also suggests that insulin resistance in Dahl S rats might still be mediated via defects in the insulin receptor substrates –1 and/or –2, possibly genetic mutation(s) that hinder(s) their association with downstream molecules in the insulin signaling pathway and prevent(s) glucose uptake by the cells.

In conclusion, we can learn a great deal from studies on insulin resistance in Dahl S rats, however the following 4 points stand out: 1) Insulin resistance in Dahl S rats precedes hypertension, 2) Insulin resistance in Dahl S rats is significantly enhanced after 4 weeks of 8% NaCl, which represents the threshold for a salt-induced suppression in the insulin signaling pathway, 3) Salt-induced insulin resistance in Dahl S rats (after 4 weeks of 8% NaCl) is accompanied by an enhanced oxidative stress and inflammatory responses that is manifested by increased levels of TNF-alpha and JNK proteins, 4) Increased TNF-alpha and JNK levels might disrupt the insulin signaling pathway at the level of the insulin receptor substrates' association with either upstream or downstream molecules in the insulin pathway or via suppressing GLUT4 expression and/or translocation. Finally, it is worth mentioning that the promoter regions of the insulin receptor substrates –1, and –2 comprises putative sodium-responsive elements that might be acted upon by excess salt to suppress their corresponding activity, expression and/or function [16,17]. Mutations in these sodium-response elements might predict protection against the impact of salt in enhancing insulin resistance in Dahl S rats. Yes, we can conclude that high salt diet contributes a great deal in advancing insulin resistance in Dahl S rats whether directly by acting on sodium-response elements in genes along the insulin signaling pathway, or indirectly by enhancing an inflammatory reaction that disrupts the insulin signaling.
